# Co-Occurrence of 12-Month Alcohol and Drug Use Disorders and Personality Disorders in the United States: Results From the National Epidemiologic Survey on Alcohol and Related Conditions

**Published:** 2006

**Authors:** Bridget F. Grant, Frederick S. Stinson, Deborah A. Dawson, S. Patricia Chou, W. June Ruan, Roger P. Pickering

**Affiliations:** Laboratory of Epidemiology and Biometry, Division of Intramural Clinical and Biological Research, National Institute on Alcohol Abuse and Alcoholism, National Institutes of Health, Bethesda, MD

## Abstract

**Background:**

Very little information is available on the co-occurrence of different personality disorders (PDs) and alcohol and drug use disorders in the U.S. population.

**Objective:**

To present national data on sex differences in the co-occurrence of Diagnostic and Statistical Manual of Mental Disorders, Fourth Edition (DSM–IV) alcohol and drug use disorders and 7 of the 10 DSM–IV PDs.

**Design:**

Face-to-face interviews conducted in the 2001–2002 National Epidemiologic Survey on Alcohol and Related Conditions (N = 43,093).

**Setting:**

The United States and the District of Columbia, including Alaska and Hawaii.

**Participants:**

Household and group-quarters residents, age 18 and older.

**Results:**

Among individuals with a current alcohol use disorder, 28.6 percent (95 percent confidence interval [CI], 26.7–30.6) had at least one PD, whereas 47.7 percent (95 percent CI, 43.9–51.6) of those with a current drug use disorder had at least one PD. Further, 16.4 percent (95 percent CI, 15.1–17.6) of individuals with at least one PD had a current alcohol use disorder, and 6.5 percent (95 percent CI, 5.7–7.3) had a current drug use disorder. Associations between PDs and alcohol and drug use disorders were overwhelmingly positive and significant (P < .05). Overall, alcohol use disorders were most strongly related to antisocial (odds ratio [OR], 4.8; 95 percent CI, 4.1–5.6), histrionic (OR, 4.7; 95 percent CI 3.8–5.8), and dependent (OR, 3.0; 95 percent CI, 1.9–4.8) PDs. Drug use disorders also were more highly associated with antisocial (OR, 11.8; 95 percent CI, 9.7–14.3), histrionic (OR, 8.0; 95 percent CI, 6.0–10.7), and dependent (OR, 11.6; 95 percent CI, 7.1–19.1) PDs. Associations between obsessive-compulsive, histrionic, schizoid, and antisocial PDs and specific alcohol and drug use disorders were significantly stronger (P < .04) among women than men, whereas the association between dependent PD and drug dependence was significantly greater (P < .04) among men than women.

**Conclusions:**

The co-occurrence of PDs with alcohol and drug use disorders is pervasive in the U.S. population. Results highlight the need for further research on the underlying structure of these disorders and the treatment implications of these disorders when comorbid.

Numerous studies have addressed the prevalence of personality disorders (PDs), especially antisocial PD, among alcohol and drug abusers.^1^ They show a high but variable rate of a broad range of PDs in alcohol and drug abusers, and several among them have demonstrated the adverse effect of these disorders on duration of stay in treatment and outcome.^2^–^9^ Studies of alcohol and drug use disorders among patients seeking treatment for personality psychopathology are rare. A recent study,^10^ however, has found high prevalences of alcohol and drug use disorders in patients seeking treatment for PDs. With few exceptions, psychiatric comorbidity in these clinical studies did not differentiate between alcohol and drug use disorders, and these studies were conducted in predominantly male samples. That this literature has paid little attention to sex differences is surprising, considering that the importance of distinguishing men and women is firmly established in the field of substance use disorder research.

From an epidemiological perspective, however, a more serious problem with research on comorbidity in clinical studies is that the samples of subjects do not represent the underlying populations. Because of this problem, it is necessary to turn to general population samples for more accurate and precise information on the comorbidity of PDs and alcohol and drug use disorders. However, large epidemiologic surveys conducted in the United States during the past two decades have focused exclusively on the prevalence and comorbidity of antisocial PD and alcohol and drug use disorders.^11^,^12^ With the exception of antisocial PD, we have very limited knowledge of the comorbidity between the range of PDs and alcohol and drug use disorders and whether these associations differ between men and women. The fact that accurate data on the sex-specific prevalences of a broad range of PDs have not been available in general population surveys of the United States reflects a major gap in our understanding of the processes underlying the comorbidity of PDs and alcohol and drug use disorders. The present study was designed, in part, to address this gap and provide the information.

Accordingly, this article presents nationally representative data on the prevalence and co-occurrence of alcohol and drug use disorders and 7 of the 10 PDs defined in the *Diagnostic and Statistical Manual of Mental Disorders, Fourth Edition* (DSM–IV)^13^ assessed in the 2001–2002 National Institute on Alcohol Abuse and Alcoholism (NIAAA) National Epidemiologic Survey on Alcohol and Related Conditions (NESARC).^14^ NESARC is the largest comorbidity survey ever conducted (*N* = 43,093). The sample size allows for accurate estimation of current or past-year co-occurrence of both alcohol and drug use disorders and avoidant, dependent, obsessive-compulsive, histrionic, paranoid, schizoid, and antisocial PDs among men and women.

## Methods

### NESARC Sample

Wave 1 of NESARC is a nationally representative face-to-face survey of 43,093 respondents, age 18 and older, conducted by NIAAA in 2001 through 2002. (A second wave will be conducted in 2004–2005.)^14^ The target population of NESARC’s Wave 1 is the civilian, noninstitutionalized population residing in the United States and the District of Columbia, including Alaska and Hawaii. The housing-unit sampling frame of NESARC was the U.S. Census Bureau Census 2000 Supplementary Survey,^14^ a national survey of more than 78,000 households per month conducted in 2000 through 2001. NESARC also included a group-quarters sampling frame derived from the Census 2000 Group Quarters Inventory.^14^ The group-quarters sampling frame captures important subgroups of the population with heavy substance use patterns (e.g., college housing) not often included in general population surveys. The sampling frame response rate was 99 percent, the household response rate was 89 percent, and the person response rate was 93 percent, yielding an overall survey response rate of 81 percent, substantially higher than other surveys of this kind.

Information on race and ethnicity collected in the Census 2000 Supplementary Survey in 2000 through 2001 was used to oversample African American and Hispanic households. The oversampling procedure increased the percentage of non-Hispanic, African American households in the sample from 12.3 percent to 19.1 percent (*N* = 8,245) and the percentage of Hispanic households from 12.5 percent to 19.3 percent (*N =* 8,308). One sample person from each household or group-quarters unit was randomly selected for interview, and young adults, ages 18–24, were oversampled at a rate of 2.25 times that of other members in the household.

NESARC data were weighted to reflect the probabilities of the selection of primary sampling units (PSUs) within strata and for the selection of housing units within the sample PSUs. The PSUs are mutually exclusive categories of persons or units of interest identified in the first stage of the multistage NESARC sample. The PSUs consisted of geographic units representing the entire United States defined in terms of sociodemographic criteria. The data also were weighted: (1) to account for the selection of one sample person from each household; (2) to account for oversampling of young adults; (3) to adjust for nonresponse at the household level and person level; and (4) to reduce the variance arising from selecting two PSUs to represent an entire stratum. The weighted data were then adjusted to be representative of the U.S. civilian, noninstitutionalized population for a variety of socioeconomic variables including region, age, sex, race, and ethnicity using the 2000 Decennial Census of Population and Housing^14^ and statistics on births, deaths, immigration and emigration, and the size of the armed forces.

### Interviewer Training and Field Quality Control

Approximately 1,800 experienced lay interviewers from the U.S. Census Bureau administered NESARC using laptop computer–assisted software that included built-in skip, logic, and consistency checks. On average, the interviewers had 5 years’ experience working on Census and other health-related national surveys. All NESARC interviewers completed a 5-day self-study at home and participated in a standardized 5-day in-class training session at one of the bureau’s 12 regional offices. NESARC training supervisors from each regional office also were required to complete the home study and to attend a centralized training session prior to fielding of the survey, where they completed the in-class training under the direction of NIAAA sponsors and Census Field and Demographics Survey Division headquarters staff.

Regional supervisors recontacted a random 10 percent of all respondents for quality-control purposes. In these quality-control interviews, a series of questions were reasked to verify that respondents had received the entire interview and that the questionnaire had been administered properly. There was no case in which it was determined that the interview had been conducted in any manner that was inconsistent with the interviewer’s extensive training. In addition, 2,657 respondents were randomly selected to participate in a reinterview study after completion of their NESARC interview. Each respondent was readministered one to three sections of the survey assessment instrument. These interviews not only served as an additional check on survey data quality but also formed the basis of a test–retest reliability study of new modules of the survey instrument.^15^

### Alcohol and Drug Use Disorder Assessment

Diagnoses presented in this article were made by the NIAAA Alcohol Use Disorder and Associated Disabilities Interview Schedule–DSM–IV Version (AUDADIS–IV),^16^ a state-of-the-art structured diagnostic interview designed to be used by lay interviewers. The AUDADIS–IV included an extensive list of symptom questions that separately operationalized DSM–IV criteria for alcohol and drug abuse and dependence including 10 classes of drugs: sedatives, tranquilizers, opiates (other than heroin or methadone), stimulants, hallucinogens, cannabis, cocaine (including crack cocaine), inhalants/solvents, heroin, and other drugs. Consistent with the DSM–IV, current (in the last 12 months) dependence diagnoses required the respondent to satisfy at least three of the seven DSM–IV criteria for dependence during the last year. The withdrawal criterion of the alcohol dependence diagnosis was measured as a syndrome, requiring at least two positive symptoms of withdrawal as defined in the DSM–IV alcohol withdrawal category. The AUDADIS–IV diagnoses of alcohol abuse required a respondent to meet at least one of the four criteria defined for abuse in the 12-month period preceding the interview and not meet criteria for dependence. The drug-specific diagnoses of abuse and dependence were derived using the same algorithm and were aggregated to produce measures of any drug use disorder, any drug abuse, and any drug dependence.

The reliability of AUDADIS–IV alcohol and drug use disorder measures was assessed in several large test–retest studies conducted in clinical and general population samples.^17^–^21^ The reliability of alcohol and drug abuse and dependence in these studies was excellent, exceeding *kappa* = 0.74 for alcohol diagnoses and *kappa* = 0.79 for drug diagnoses. The discriminant, concurrent, convergent, construct, and population validities of the AUDADIS–IV alcohol and drug use disorder diagnoses also have been well documented,^22^–^35^ including in the World Health Organization/National Institutes of Health Reliability and Validity Study.^36^–^40^ In these studies,^33^ alcohol and drug use disorder diagnoses were found to be significantly and highly correlated with important validators, including substance use, social/occupational dysfunction and disability, and family history (convergent validity), 24–26,32–34 and these results were shown to generalize to other populations (population validity).^27^ These studies also demonstrated that abuse and dependence diagnoses also were related to different sets of validators (discriminant validity). Alcohol and drug use disorder diagnoses defined by *Diagnostic and Statistical Manual of Mental Disorders, Third Edition* (DSM–III), *Diagnostic and Statistical Manual of Mental Disorders, Third Edition, Revised* (DSM–III–R), DSM–IV, and *International Classification of Diseases, 10th Revision* (ICD–10)^41^ criteria also were shown to be highly concordant (convergent validity).^22^,^23^,^28^–^30^,^37^ Concordance between AUDADIS–IV alcohol and drug use disorders and those assessed with the *Schedule for Clinical Assessment in Neuropsychiatry*^42^ was high (concurrent validity),^36^,^39^ and the construct validity of these diagnoses has been supported by both exploratory and confirmatory factor analyses.^32^,^35^,^38^

### Personality Disorder Assessment

The diagnosis of PDs requires an evaluation of the individual’s long-term patterns of functioning.^13^^(p630)^ Diagnoses of PDs made using the AUDADIS–IV were made accordingly. Respondents were asked a series of personality symptom questions about how they felt or acted most of the time throughout their lives regardless of the situation or whom they were with. They were reminded on 20 occasions throughout the PD section not to include times when they were depressed, manic, anxious, drinking heavily, using medicines or drugs, experiencing withdrawal symptoms (defined earlier in the AUDADIS–IV), or times when they were physically ill.

To receive a DSM–IV diagnosis, respondents needed to endorse the requisite number of DSM–IV symptom items for the particular PD, and at least one positive symptom item must have caused social or occupational dysfunction. Multiple symptom items were used to operationalize the more complex criteria associated with certain PDs. The following numbers of symptom items were used to assess each PD: avoidant (*N* = 7); dependent (*N* = 8); obsessive-compulsive (*N* = 10); paranoid (*N* = 9); schizoid (*N* = 10); histrionic (*N* = 11); and antisocial (*N* = 30). Because of time and space constraints, not all DSM–IV PDs were assessed in Wave 1 NESARC. The decision to exclude borderline, schizotypal, and narcissistic PDs was based on the larger number of symptom items required to operationalize the disorders relative to those PDs assessed in Wave 1 (i.e., borderline, 18 items; schizotypal, 16 items; and narcissistic, 19 items). However, in the follow-up Wave 2 of NESARC, borderline, schizotypal, and narcissistic PDs will be included.

The reliability of AUDADIS–IV PDs was assessed in a test–retest study conducted as part of the NESARC survey proper.^15^ A random subsample of 282 respondents was reinterviewed with the antisocial PD module, and another subsample of 315 respondents was reinterviewed with the AUDADIS–IV modules containing the remaining PD measures. These reinterviews were conducted approximately 10 weeks after the NESARC interviews. The reliability of the PD diagnoses in these community samples ranged from fair to good, from *kappa* = 0.40 for histrionic PD to *kappa* = 0.67 for antisocial PD. Reliabilities of the AUDADIS–IV PD diagnoses are as good as or better than those found for semistructured personality interviews in short-term test–retest studies conducted in treated samples of patients.^43^

The validity of AUDADIS–IV PDs was assessed in a series of linear regression analyses, using NESARC data, that examined the associations between each PD and three Short Form 12v2^44^ disability scores, controlling for age, all other PDs, and 12-month comorbid DSM–IV substance use disorders and anxiety and mood disorders. The Short Form 12v2, a reliable and valid measure of generic quality of life used in large population surveys, yields 10 component summary and profile scores assessing various dimensions of disability and impairment. In the present analyses, the focus was on three Short Form 12v2 scores: the mental component summary score; the social functioning score, reflecting limitations in social functioning; and the role emotional function score, measuring role impairment due to emotional problems. All PDs, except histrionic, were shown to be highly significant (*P* < .01 to *P* < .001) predictors of the mental component summary, social functioning, and role emotional scores. Respondents with those PDs had significantly greater disability and social/occupational dysfunction than respondents who did not have the PD.

### Statistical Analysis

Cross-tabulations were used to calculate prevalences and comorbidity rates of PDs and alcohol and drug use disorders. A series of univariate logistic regression analyses were used to study associations between PDs and alcohol and drug use disorders. The *beta* coefficients from these analyses were transformed into odds ratios (ORs) for ease of interpretation. Differences in the associations of PDs and alcohol and drug use disorders between men and women were examined by comparing sex-specific *beta* coefficients derived from the logistic regression analyses. Because of the complex survey design of NESARC, variance estimation procedures that assume simple random sampling cannot be used. The stratification of the NESARC sample will result in standard errors much larger than those that would be obtained with a simple random sample of equal size. To take into account this NESARC sample design component, all standard errors and 95 percent confidence limits (CIs) presented here were generated using SUDAAN (Software for Survey Data Analysis),^45^ a software program that uses appropriate statistical techniques to adjust for sample design characteristics.

## Results

### Prevalence of Alcohol and Drug Use Disorders and PDs

The 12-month prevalences of any alcohol use disorder and any drug use disorder were 8.5 percent and 2.0 percent, respectively (Table 1). Rates of abuse exceeded those for dependence for both alcohol and drug use disorders. The most prevalent PD in the general population was obsessive-compulsive PD (7.9 percent), followed by paranoid PD (4.4 percent), antisocial PD (3.6 percent), schizoid PD (3.1 percent), avoidant PD (2.4 percent), histrionic PD (1.8 percent), and dependent PD (0.5 percent).

### Prevalence of PDs Among Respondents With 12-Month Alcohol and Drug Use Disorders

As indicated in the top row of Table 2, 28.6 percent and 47.7 percent of respondents with a 12-month alcohol use disorder and drug use disorder, respectively, had at least one PD. Rates of any PD were greater among respondents with any drug abuse (37.8 percent) and any drug dependence (69.5 percent) than among respondents with alcohol abuse (19.8 percent) and alcohol dependence (39.5 percent). The prevalence of antisocial PD (12.3 percent), obsessive-compulsive PD (12.1 percent), and paranoid PD (10.2 percent) were the highest among respondents with an alcohol use disorder. These also were the most prevalent PDs among respondents with any drug use disorder, but the rates were much higher. The prevalence of specific PDs was much greater among respondents with dependence on alcohol (2.5 percent–18.3 percent) or drugs (10.1 percent–39.5 percent) compared with respondents with alcohol abuse (0.3 percent–9.5 percent) or any drug abuse (2.0 percent–22.3 percent).

### Prevalence of 12-Month Alcohol and Drug Use Disorders Among Respondents With PDs

As indicated in Table 3, 16.4 percent of the respondents with at least one PD met criteria for a current alcohol use disorder, and 6.5 percent met criteria for a current drug use disorder. The prevalence of any alcohol use disorder was greatest among respondents with histrionic (29.1 percent), antisocial (28.7 percent), dependent (21.6 percent), and paranoid (19.5 percent) PDs. Similarly, the rate of any drug use disorder was greatest among respondents with dependent (18.5 percent), antisocial (15.2 percent), and histrionic (12.8 percent) PDs. Prevalences of alcohol abuse (2.5 percent–9.5 percent) and drug abuse (2.0 percent–8.4 percent) were consistently lower among respondents with specific PDs than the corresponding rates for alcohol dependence (7.4 percent–21.3 percent) and any drug dependence (2.3 percent–12.9 percent). The only exception to this pattern was among respondents with antisocial PD, where the prevalence of any drug abuse (8.4 percent) exceeded the rate for any drug dependence (6.8 percent).

### Associations Between Alcohol and Drug Use Disorders and PDs

Associations between alcohol and drug use disorders and PDs are shown in Table 4 in the form of ORs. The overall pattern of ORs is overwhelmingly positive, with 88 percent of the disorder-specific ORs being positive and statistically significant. The association between any PD and any alcohol use disorder (OR, 2.6) was weaker than the association found for any drug use disorder (OR, 5.5), a pattern also found when specific PDs were examined. Specific PDs were more strongly related to alcohol dependence (ORs, 2.2–7.5) and drug dependence (ORs, 4.8–26.0) than to alcohol abuse (ORs, 0.5–2.2) or drug abuse (ORs, 1.5–8.2). Although histrionic PD (OR, 1.7) and antisocial PD (OR, 2.2) were significantly associated with alcohol abuse, the associations between alcohol abuse and avoidant, dependent, obsessive-compulsive, paranoid, and schizoid PDs were not significant. All specific PDs, however, were strongly and consistently related to any alcohol use disorder (ORs, 1.7–4.8) and any drug use disorder (ORs, 2.4–11.8). Dependent, histrionic, and antisocial PDs were more strongly related to both alcohol and drug use disorders than any of the other PDs.

### Associations Between Alcohol and Drug Use Disorders and PDs, by Sex

Similar to the pattern observed in the total sample, the associations between current alcohol and drug use disorders and PDs among men and women were overwhelmingly significant and positive, with the exception of the associations between avoidant, dependent, obsessive-compulsive, paranoid, and schizoid PDs and alcohol abuse (Table 5). With respect to any drug use disorder, drug abuse, and drug dependence, associations remained the strongest for antisocial, histrionic, and dependent PDs among men and women. The same pattern was observed for any alcohol use disorder and alcohol dependence among men and women.

Significant sex differences in the associations between alcohol and drug use disorders and PDs also were observed. The relationship between obsessive-compulsive (*P* < .02), histrionic (*P* < .04), and antisocial (*P* < .006) PDs and alcohol dependence was significantly greater for women than men. With regard to any drug abuse, the associations with obsessive-compulsive (*P* < .03), schizoid (*P* < .009), histrionic (*P* < .02), and antisocial (*P* < .002) PDs were greater for women than for men. In contrast, the association between drug dependence and dependent PD was significantly greater (*P* < .04) among men than women.

## Comment

The co-occurrence of DSM–IV current alcohol and drug use disorders and DSM–IV PDs is pervasive in the U.S. population. Among individuals with a current alcohol or drug use disorder, 28.6 percent and 47.7 percent, respectively, had at least one PD. While the proportion of individuals with a PD who also had an alcohol or drug use disorder was lower, a considerable proportion of those with PDs did meet criteria for alcohol or drug abuse or dependence. Overall, 16.4 percent of individuals in the general population with at least one PD had a current alcohol use disorder, and 6.5 percent had a current drug use disorder. The strong associations between most PDs and alcohol and drug use disorders were generally consistent when examined separately among men and women. Consistent with clinical research on comorbidity of Axis II disorders and alcohol and drug use disorders,^1^ this study found greater associations between PDs and drug use disorders compared with alcohol use disorders.

Comorbidity in the general population is often lower than comorbidity in treated samples since individuals with more than one disorder have a greater probability of seeking treatment (i.e., Berkson bias). However, a striking finding in this study was that the prevalence of any PD and antisocial PD (one of the most extensively studied PDs in treated samples) among individuals with current alcohol and drug use disorders was similar to the median rates observed in samples of patients receiving treatment for alcohol and/or drug use disorders, as assessed with other standardized assessment instruments (i.e., the Structured Clinical Interview for DSM–III–R Personality Disorders [SCID–II]^46^ and the Diagnostic Interview Schedule^47^). For example, the median rate of any PD among patients receiving treatment for an alcohol use disorder assessed with the SCID–II^48^,^49^ was 39.0 percent compared with 39.5 percent found among individuals in this study with current alcohol dependence. The median rate of any PD among patients receiving treatment for drug use using the SCID–II 4,7,10,49–57 was 59.0 percent compared with the 69.5 percent rate found among individuals with current drug dependence. The prevalence of antisocial PD among respondents with current drug dependence was 39.5 percent, a figure midway between the median rates found in studies of drug treatment samples using the semistructured SCID–II^50^,^52^,^53^,^55^,^58^–^60^ (21.0 percent) and the fully structured Diagnostic Interview Schedule^61^–^64^ (49.0 percent) assessment instruments. The rate of antisocial PD among individuals with current alcohol dependence was 18.3 percent, somewhat lower than the median rate of 37.5 percent found among patients in alcohol treatment settings using the Diagnostic Interview Schedule.^65^–^72^ It is likely that the prevalences of PDs among individuals with alcohol and drug use disorders in this study would have been greater if all DSM–IV PDs had been assessed. If all PDs had been assessed, we might expect the reported rates of PDs using the fully structured AUDADIS–IV to have slightly exceeded the rates presented earlier for semistructured interviews, as would be predicted by the literature.

The PDs most strongly associated with alcohol and drug use disorders were antisocial, dependent, and histrionic PDs. The degree of diagnostic overlap between DSM–III PDs has long been recognized,^73^,^74^ and it may be responsible for the strong relationship observed between histrionic, antisocial, and dependent PDs and alcohol and drug use disorders. For example, individuals with antisocial PD share certain tendencies with individuals with histrionic PD to be impulsive, seductive, superficial, excitement seeking, reckless, and manipulative, but individuals with histrionic PD do not characteristically exhibit antisocial behaviors.^13^ Individuals with dependent PD and histrionic PD are excessively dependent on others for praise, guidance, and nurturance, but individuals with dependent PD do not characteristically demonstrate the flamboyant emotional features of histrionic PD. Although multivariate studies^75^–^77^ have been conducted on itemlevel criteria of DSM PDs in search of the factor structure underlying PD diagnoses, the findings of this study suggest that this search be expanded to include criteria of Axis I substance use disorders along with the components of PDs that are most closely associated with them. The results of these future studies might elucidate subtypes of alcohol and drug use disorders, refine the classification of both types of disorder, and increase our understanding of the pathological processes underlying their comorbidity.

A number of the PDs examined in this study were more strongly associated with alcohol and drug use disorders among women, including antisocial PD. However, a stronger association between dependent PD and drug dependence was observed among men. Although reasons for these observed sex differences are unknown, these findings highlight the need to examine a broader set of factors that affect the prevalence and co-occurrence of PDs and alcohol and drug use disorders, including age, socioeconomic status, and, importantly, primary substance of abuse. In the current study, the stronger associations observed between antisocial PD and alcohol and drug use disorders among women may be the result of differential mortality or incarceration. That is, men who are highly comorbid for antisocial PD and alcohol and drug use disorders are more likely to die young or be incarcerated than women and thus less likely to be represented in general population surveys. This explanation is consistent with the findings that men are overrepresented in jail and prison populations and that substance use disorders occur in about 90 percent of individuals with antisocial PD who are incarcerated.^78^

In light of the extensive comorbidity between PDs and alcohol and drug use disorders found in this study, there would appear to be great value in assessing a broad range of PDs among substance abuse patients. This more comprehensive assessment can guide treatment planning. For example, patients with comorbid alcohol and drug use disorders and PDs can be expected to require treatment that is more extensive and of longer duration. In this regard, modified psychoanalytic psychotherapy focused or targeted on particular features of PDs might hold great promise for successful recovery among comorbid individuals. 79–81 The trend toward integrating 12-step programs into rehabilitation programs also appears promising in that 12-step programs require individuals to examine their relationship to others, overcome feelings of helplessness, gain an internal locus of control, encourage self-examination, address defects in character, and promote honest relationships. 82,83 More clinical research is needed to examine the role of these and other approaches targeted at treating substance use disorders (e.g., contingency management, motivational enhancement therapy, cognitive behavior therapy) in improving the chances of recovery and the lives of individuals with comorbid alcohol and drug use disorders and PDs. This work will be formidable, because some of these components of treatment are on uncertain grounds in terms of efficacy and mechanisms of action (e.g., 12-step programs and psychoanalytic treatments). Attention in this clinical work on the effects of sex, substance of choice, and other factors that affect treatment outcome and eventual recovery might further refine treatment planning.

This national study of comorbidity represents a landmark study in the area of PDs. Previous psychiatric epidemiology studies were too small to address these important relationships in detail. Personality disorders are not only pervasive and associated with substantial disability,^44^ they are very common among those with alcohol and drug use disorders. Further work in many directions is indicated by the results of this study, including a dissection of the components of the two types of disorders that are most closely associated, the factors giving rise to the associations, and the treatment and prevention implications of these disorders when comorbid.

## Figures and Tables

**Table 1:** Twelve-Month Prevalence of Alcohol and Drug Use Disorders and Prevalence of Personality Disorders (PDs)

Disorder	Prevalence		No. of Subjects†
	%	(S.E.)*	
Any alcohol use disorder	8.5	(0.24)	3327
Alcohol abuse	4.7	(0.18)	1843
Alcohol dependence	3.8	(0.14)	1484
Any drug use disorder	2.0	(0.10)	777
Any drug abuse	1.4	(0.08)	528
Any drug dependence	0.6	(0.05)	249
Any PD	14.8	(0.36)	6295
Avoidant PD	2.4	(0.11)	995
Dependent PD	0.5	(0.05)	208
Obsessive–compulsive PD	7.9	(0.23)	3261
Paranoid PD	4.4	(0.15)	2105
Schizoid PD	3.1	(0.12)	1425
Histrionic PD	1.8	(0.09)	808
Antisocial PD	3.6	(0.15)	1422

* Prevalence figures are based on weighted data.

† Number of subjects is based on unweighted data.

**Table 2:** Prevalence of Personality Disorders (PDs) Among Respondents With a 12-Month Alcohol or Drug Use Disorder*

Comorbid Disorder	Alcohol			Any Drug		
	
	Disorder	Abuse	Dependence	Disorder	Abuse	Dependence
Any PD	28.6 (1.00)	19.8 (1.16)	39.5 (1.63)	47.7 (1.96)	37.8 (2.35)	69.5 (3.30)
Avoidant PD	4.5 (0.43)	2.0 (0.37)	7.7 (0.86)	10.1 (1.35)	6.5 (1.38)	18.2 (3.56)
Dependent PD	1.3 (0.25)	0.3 (0.13)	2.5 (0.54)	4.6 (1.06)	2.0 (0.63)	10.1 (3.01)
Obsessive–compulsive PD	12.1 (0.69)	9.5 (0.90)	15.2 (1.10)	16.9 (1.66)	11.5 (1.60)	28.7 (3.80)
Paranoid PD	10.2 (0.61)	5.6 (0.74)	15.8 (1.13)	18.6 (1.73)	11.9 (1.57)	33.2 (4.03)
Schizoid PD	5.1 (0.51)	2.5 (0.40)	8.2 (0.98)	12.3 (1.48)	8.4 (1.41)	21.0 (3.39)
Histrionic PD	6.3 (0.51)	3.1 (0.47)	10.3 (0.95)	11.8 (1.47)	7.8 (1.24)	20.6 (3.63)
Antisocial PD	12.3 (0.72)	7.4 (0.74)	18.3 (1.22)	27.7 (1.76)	22.3 (2.25)	39.5 (3.55)

* Values are expressed as percentage (SE).

**Table 3:** Prevalence of 12-Month Alcohol and Drug Use Disorders Among Respondents With a Personality Disorder (PD)*

Comorbid Disorder	Any PD	Avoidant	Dependent	Obsessive-compulsive	Paranoid	Schizoid	Histrionic	Antisocial
Any alcohol use disorder	16.4 (0.62)	16.3 (1.43)	21.6 (3.97)	12.9 (0.77)	19.5 (1.17)	13.7 (1.32)	29.1 (2.19)	28.7 (1.53)
Alcohol abuse	6.2 (0.38)	3.9 (0.71)	2.5 (1.20)	5.6 (0.54)	5.9 (0.77)	3.7 (0.59)	7.8 (1.19)	9.5 (0.94)
Alcohol dependence	10.2 (0.51)	12.4 (1.33)	19.1 (3.93)	7.4 (0.60)	13.6 (1.04)	9.9 (1.17)	21.3 (1.88)	19.2 (1.29)
Any drug use disorder	6.5 (0.41)	8.6 (1.17)	18.5 (3.73)	4.3 (0.48)	8.4 (0.87)	7.9 (1.06)	12.8 (1.59)	15.2 (1.16)
Any drug abuse	3.5 (0.29)	3.8 (0.82)	5.7 (1.71)	2.0 (0.29)	3.7 (0.52)	3.7 (0.66)	5.8 (0.96)	8.4 (0.97)
Any drug dependence	2.9 (0.28)	4.8 (1.03)	12.9 (3.69)	2.3 (0.38)	4.7 (0.71)	4.2 (0.77)	7.0 (1.35)	6.8 (0.87)

* Values are expressed as percentage (SE).

**Table 4:** Odds Ratio (ORs) of Personality Disorders (PDs) and Alcohol and Drug Use Disorders*

Comorbid Disorder	Alcohol†			Any Drug†		
	
	Disorder	Abuse	Dependence	Disorder	Abuse	Dependence
Any PD	2.6 (2.3–2.8)	1.4 (1.2–1.7)	4.0 (3.6–4.6)	5.5 (4.7–6.5)	3.5 (2.9–4.4)	13.5 (9.9–18.2)
Avoidant PD	2.2 (1.7–2.7)	0.8 (0.6–1.2)	3.8 (3.0–4.9)	5.0 (3.7–6.8)	2.9 (1.9–4.7)	9.6 (5.0–15.6)
Dependent PD	3.0 (1.9–4.8)	0.5 (0.2–1.4)	6.1 (3.6–10.1)	11.6 (7.1–19.1)	4.4 (2.3–8.5)	26.0 (13.3–50.6)
Obsessive–compulsive PD	1.7 (1.5–1.9)	1.2 (1.0–1.5)	2.2 (1.8–2.6)	2.4 (1.9–3.1)	1.5 (1.1–2.1)	4.8 (3.3–6.9)
Paranoid PD	2.8 (2.4–3.3)	1.3 (1.0–1.7)	4.6 (3.8–5.5)	5.3 (4.2–6.7)	3.0 (2.2–4.1)	11.3 (7.8–16.2)
Schizoid PD	1.7 (1.4–2.2)	0.8 (0.6–1.1)	2.9 (2.3–3.9)	4.6 (3.5–6.2)	2.9 (2.0–4.2)	8.6 (5.7–13.0)
Histrionic PD	4.7 (3.8–5.8)	1.7 (1.2–2.5)	7.5 (6.0–9.4)	8.0 (6.0–10.7)	4.7 (3.3–6.8)	14.8 (9.5–23.0)
Antisocial PD	4.8 (4.1–5.6)	2.2 (1.8–2.8)	7.1 (6.0–8.4)	11.8 (9.7–14.3)	8.2 (6.2–10.9)	18.5 (13.6–25.1)

* ORs represent the odds of having a PD among individuals with a specific alcohol or drug use disorder relative to the odds of having a PD among individuals who do not have the specific alcohol or drug use disorder.

† Values are expressed as OR (95% confidence interval).

**Table 5:** Twelve-Month Odds Ratios (ORs) of Personality Disorders (PDs) and Alcohol and Drug Use Disorders by Sex*

Comorbid Disorder	Alcohol†			Any Drug†		
	
	Disorder	Abuse	Dependence	Disorder	Abuse	Dependence
**Men**						
Avoidant PD	2.4 (1.7–3.3)	0.8 (0.5–1.5)	4.3 (3.0–6.1)	5.7 (3.6–8.8)	2.9 (1.4–5.7)	11.8 (6.3–22.0)
Dependent PD	4.4 (2.0–9.7)	0.5 (0.1–2.2)	9.3 (4.1–21.4)	17.1 (8.2–35.9)	2.7 (0.8–9.0)	48.4 (20.8–113.5)‡
Obsessive–compulsive PD	1.6 (1.3–1.9)	1.3 (1.0–1.7)	1.9 (1.5–2.3)	2.3 (1.6–3.1)	1.2 (0.8–1.8)	5.2 (3.3–8.5)
Paranoid PD	3.1 (2.6–3.9)	1.2 (0.8–1.9)	5.3 (4.1–6.8)	5.4 (3.9–7.5)	2.7 (1.7–4.4)	12.4 (7.5–20.6)
Schizoid PD	1.6 (1.2–2.1)	0.6 (0.4–1.0)	2.9 (2.1–4.0)	3.9 (2.7–5.7)	1.9 (1.0–3.3)	9.0 (5.3–15.3)
Histrionic PD	4.3 (3.2–5.8)	1.8–(1.1–2.7)	6.4 (4.7–8.7)	7.2 (4.9–10.6)	3.2 (1.8–5.5)	16.4 (9.5–28.6)
Antisocial PD	3.5 (2.9–4.2)	1.6 (1.3–2.2)	5.3 (4.4–6.5)	8.5 (6.6–10.9)	5.6 (4.1–7.8)	14.8 (9.7–22.4)
**Women**						
Avoidant PD	2.5 (1.8–3.5)	1.1 (0.6–1.8)	4.1 (2.9–5.9)	5.3 (3.5–8.1)	3.7 (2.1–6.6)	8.6 (4.8–15.6)
Dependent PD	2.8 (1.6–4.9)	0.8 (0.2–2.4)	5.1 (2.7–9.4)	10.1 (5.4–19.0)	7.9 (3.5–17.8)	13.4 (5.7–31.5)
Obsessive–compulsive PD	2.0 (1.5–2.5)	1.1 (0.8–1.6)	2.9 (2.2–4.0)§	2.9 (2.0–4.1)	2.3 (1.4–3.8)§	4.0 (2.3–6.9)
Paranoid PD	3.0 (2.4–3.8)	1.7 (1.2–2.4)	4.5 (3.4–6.0)	6.2 (4.5–8.4)	4.0 (2.6–6.3)	11.4 (7.1–18.3)
Schizoid PD	2.1 (1.5–2.9)	1.1 (0.7–1.9)	3.1 (2.1–4.6)	6.3 (4.3–9.1)	5.4 (3.2–9.0)§	7.8 (4.4–13.9)
Histrionic PD	5.8 (4.3–7.7)	1.7 (1.0–2.9)	10.2 (7.4–14.0)§	9.7 (6.3–15.0)	8.1 (4.8–13.8)§	11.9 (6.4–21.9)
Antisocial PD	6.2 (4.7–8.2)	2.7 (1.8–4.1)	9.4 (8.7–13.2)§	17.9 (12.5–25.5)	14.0 (8.8–22.3)§	22.6 (13.4–38.2)

* ORs represent the odds of having a PD among individuals with a specific alcohol or drug use disorder relative to the odds of having a PD among individuals who do not have the specific alcohol or drug use disorder.

† Values are expressed as OR (95% confidence interval).

‡ Indicates association significantly greater (*P* < .04) among men than women.

§ Indicates association significantly greater (*P* < .04) among women than men.
